# Effects of OnabotulinumtoxinA on Allodynia and Interictal Burden of Patients with Chronic Migraine

**DOI:** 10.3390/toxins16020106

**Published:** 2024-02-15

**Authors:** Andreas A. Argyriou, Emmanouil V. Dermitzakis, Dimitrios Rikos, Georgia Xiromerisiou, Panagiotis Soldatos, Pantelis Litsardopoulos, Michail Vikelis

**Affiliations:** 1 Headache Outpatient Clinic, Department of Neurology, Agios Andreas General Hospital of Patras, 26335 Patras, Greece; pantelis84@hotmail.com; 2 Euromedica General Clinic, 54645 Thessaloniki, Greece; manolis.dermitzakis@gmail.com; 3 404 Military Hospital, 41222 Larisa, Greece; rikosd@hotmail.com; 4 Department of Neurology, University Hospital of Larissa, University of Thessaly, 41110 Larissa, Greece; georgiaxiromerisiou@gmail.com; 5 Independent Researcher, 24100 Kalamata, Greece; soldatosp@gmail.com; 6 Headache Clinic, Mediterraneo Hospital, 16675 Athens, Greece; mvikelis@headaches.gr

**Keywords:** chronic migraine, OnabotulinumtoxinA, BoNTA, effects, interictal burden, cutaneous allodynia

## Abstract

Background: We primarily aimed to ascertain whether treatment with OnabotulinumtoxinA (BoNTA) might influence the extent of the interictal burden and cutaneous allodynia in patients with chronic migraine (CM). Methods: Seventy CM patients, who received three consecutive cycles of BoNTA, were studied. The interictal burden was assessed with the Migraine Interictal Burden Scale (MIBS-4), while cutaneous allodynia was examined with the Allodynia Symptom Checklist (ASC-12) together with PI-NRS VAS to obtain hair brushing scores, and then these were compared from baseline (T0) to the last efficacy evaluation follow-up (T1). Efficacy outcomes, mostly mean headache days (MHD) and “Headache Impact Test” scores, were also assessed between T0 and T1. Results: BONTA improved the interictal burden, with a decrease in MIBS-4 scoring by an average of −7 at T1, compared to baseline (*p <* 0.001). The percentage of patients with a moderate/severe interictal burden was substantially decreased. Likewise, BoNTA reduced the extent of cutaneous allodynia, with a significant reduction in both the ASC-12 (1 vs. 6; *p <* 0.001) and PI-NRS VAS (1 vs. 5; *p <* 0.001) to hair brushing median scores at T1, compared to baseline. Reduced MHD rates were significantly associated with a smaller interictal burden at T1. The efficacy of BoNTA, with a significant reduction in MHD and HIT-6 scores at T1 compared to T0, was re-confirmed. Conclusions: BoNTA resulted in a statistically significant reduction in the interictal burden and also improved cutaneous allodynia. The reduction in ictal burden was associated with the down-scaling of the interictal burden. Hence, BoNTA improved the full spectrum of migraine impairment by diminishing the clinical expression of central sensitization.

## 1. Introduction

Tension-type headache (TTH) and migraine are the most common primary headaches, accounting for almost 80% of all headache disorders. The remaining 20% are classified as secondary headaches due to an underlying medical condition, mostly with infectious, inflammatory, vascular, traumatic or structural causes [1]. According to the latest estimates of the Global Burden of Disease Study (GBD) in 2019, primary headache disorders, including TTH, migraine, trigeminal autonomic cephalalgias and cluster headaches, frequently occur and account for 8% of the total years lived with a disability, corresponding to about 800 million incident cases and 2.6 billion prevalent cases [2]. 

Although migraine is less common (with a prevalence of about 15% in the general population of western countries and as the seventh most common disease worldwide) than tension-type headache, it is much more disabling, causing it to rank in the top three most burdensome of all medical conditions and to account for 4.9% of the all-age global population’s ill health, quantified in years lived with a disability [2]. Migraine has a polymorphic phenotype, as, according to its different phases, it is clinically characterized by sudden attacks of moderate or severe head pain, which can be accompanied by various other symptoms preceding, overlapping or occurring within evolving migraine phases, including the prodromal phase, aura phase, ictal phase and postictal phase, with the resolution of symptoms [3,4]. 

For clinical but also for research practice purposes, migraine is classified, based on the frequency of monthly headache days (MHD), into two major forms, according to the 2018 criteria of the International Classification of Headaches Disorders III [5]. The first is episodic migraine (less than 15 MHD), which is further subcategorized into very-low- (1–3 MHD), low- (4–7 MHD) and high-frequency (8–14 MHD) episodic migraines. Second, chronic migraine (CM) is defined as headaches occurring on at least 15 days per month, with eight of these having typical migrainous features or being triptans-responsive, for more than 3 months. It is estimated that about 3% of migraineurs yearly progress from episodic migraine to CM, while the risk factors for migraine chronification include an inadequate response to acute medications and preventatives, medication overuse headache (MOH) and evidence of comorbidities—mainly psychiatric—as well as a history of neck/head trauma [6]. Although high-frequency episodic migraine (HFEM) might pose a significant burden to patients’ quality of life [7], it is generally accepted that CM is much more disabling and burdensome compared to HFEM, due to its much more complex clinical phenotype, characterized by a greater pain intensity, longer average duration of headaches and higher pain-related comorbidities, which eventually lead to substantially increased disability and less productivity in all domains of daily living [8,9]. CM generally requires much higher healthcare resource utilization due to its significantly increased direct and indirect health costs compared to either sub-category of episodic migraine [10]. 

During a migraine attack, apart from a headache, several other hypersensitivity symptoms may frequently occur and can be self-rated by patients as being more disabling than the headache itself. Common migraine-associated symptoms include osmophobia, phonophobia, photophobia, nausea/vomiting and cutaneous allodynia [11,12]. Specifically, cutaneous allodynia, ranking highly among the established key risk factors for migraine progression due to central sensitization [13,14,15], significantly heightens the disability associated with migraine, especially in patients with CM; this is mostly due the reduced likelihood of an adequate response to acute migraine therapies, including triptans [16]. Generally, cutaneous allodynia is defined as the production of a painful response resulting from the application of non-noxious stimuli to normal skin, and it is usually classified by its presence in the cephalic or extracephalic areas, as well as according to the sensory modality used to elicit it, i.e., thermal and mechanical allodynia [13,14,15]. Although the negative impact of migraine attacks (ictal phase) on patients’ daily living and quality of life has previously been well documented [17], little attention has been specifically paid to the effect of the burden of disease between migraine attacks (interictal burden) on the domains of patients’ quality of life, including productivity, cognitive ability and psychosocial as well as emotional well-being [18,19,20]. As such, there is an obvious clinical need for the prompt and effective pharmacological inhibition of central sensitization in CM to alleviate the ictal and interictal burden of the disease, as well as to reduce the extent of the burdensome symptoms accompanying headache, including cutaneous allodynia. Towards the latter view, the effect of available oral or injectable preventative medications on the interictal burden of migraine has been scarcely addressed.

OnabotulinumtoxinA (BoNTA) was approved and included among the established treatment options for CM prophylaxis in adults following the release of the pooled analysis of the PREEMPT (Phase III REsearch Evaluating Migraine Prophylaxis Therapy) trials, which showed an excellent efficacy/safety profile [21,22]. From a mechanistic point of view, the efficacy of BoNTA in significantly reducing the frequency of MHD is based on its ability to directly inhibit peripheral sensitization and neurogenic inflammation but also to indirectly suppress the central sensitization of the sensory neurons associated with cutaneous allodynia, through the blockage of glutamate, calcitonin gene-related peptide (CGRP) and substance P from the sensory nerve terminals [23]. Several real-world studies demonstrate the favorable benefit–risk ratio of BoNTA in the clinical setting of CM prophylaxis [24]. Our group has previously confirmed that BoNTA has a favorable benefit–risk ratio, when commenced in Greek CM patients with or without medication overuse headache (MOH), while our long-term data have demonstrated significantly high adherence to BoNTA at up to 3 and 5 years of continuous treatment, coupled with evidence of sustained improvements in all headache efficacy variables [25,26,27]. 

There is scarce evidence in the literature on whether BoNTA can directly exert beneficial effects in reducing the interictal burden [28] and the extent of cutaneous allodynia [29] in the setting of CM prophylaxis. As such, the primary objective of the current study was to ascertain whether treatment with three (one per trimester) consecutive BoNTA cycles might beneficially influence the interictal burden of the disease and to explore the effects of BoNTA on the frequency and extent of cutaneous allodynia in patients with CM. 

## 2. Results

### 2.1. Characteristics of the Study Sample

A total of 70 consecutive BoNTA-naïve CM patients, scheduled to receive quarterly at least three consecutive BoNTA courses, were initially enrolled, and all (100%) of them achieved treatment with the third BoNTA course. The mean age of the included patients was 44.4 ± 9.8 (range: 21–68) years, and the overwhelming majority were females (n = 65; 91.5%). The time since migraine diagnosis was a median of 16 years, while participants had failed a median number of four (range: 3–6) previous oral prophylactic medications;—either antiepileptics, antidepressants or beta or calcium channel blockers. A significant percentage of patients (n = 58; 82.6%) had coexistent MOH at baseline, while psychiatric comorbidities were disclosed in 40 patients (57.2%). Among these 40 patients, 19 (27.1%) had anxiety disorder; 12 (17.1%) had depression; 6 (8.6%) had mixed anxiety and depression disorder; and 3 (4.3%) had bipolar disorder. Migraine with an aura was not commonly seen (n = 7; 10%). Table 1 summarizes the baseline epidemiological and clinical characteristics of the participants. 

### 2.2. Effects of BoNTA on Interictal Burden and Allodynia Outcomes (Primary Objectives)

We observed significant improvements in the migraine-related interictal burden and allodynia measures, as evidenced by a strong decline in MIBS-4 (decreased by a median of −7) and ASC12 (decreased by a median of −5) scoring after three courses of BoNTA treatment at T1, compared to baseline. The severity of the interictal burden, according to MIBS-4 scoring, was moderate and severe in 2 and 68 patients, respectively, at baseline, while significant improvement in its grading was observed at T1 to account for 41 patients with mild; 4 with moderate; and 20 with a severe interictal burden. Notably, five patients with a super-response to BoNTA reported zero scoring at T1, in line with no interictal burden. Comparable beneficial effects were seen with allodynia, which was numerically disclosed in 48 (68.6%) patients at baseline and 28 (40%) patients at T1 (*p* < 0.001), strongly suggesting that BoNTA could provide complete relief from allodynia in about half of the affected patients. PI-NRS median scores for hair brushing dropped from 5 at T0 to 1 at T1 (*p* < 0.001). A summary of the changes in interictal burden and allodynia outcomes between T0 and T1 (median, range, z, *p* and effect size values) is provided in Table 2. 

The improvements in the ASC-12 and MIBS-4 scores at T1 remained unrelated (Pearson’s correlation: 0.104; *p* = 0.403), clearly demonstrating that the lessened interictal burden of our patients was not because of the beneficial effects of BoNTA on the severity of cutaneous allodynia. 

### 2.3. Responder Rates and Effects of BoNTA on Efficacy and Disability Outcomes (Secondary Objectives)

Most treated patients were classified as treatment responders (54/70; 77.2%) at T1, because they experienced at least a 50% reduction in their MHD. Among 54 responders, 35 (49.3) and 19 (26.8) maintained reductions of 50% and 75%, respectively, in their MHD at T1, compared to T0. These 54 treatment responders converted from CM to EM and from MOH to no MOH. A total of 16 (22.5%) patients failed to adequately respond to BoNTA treatment, experiencing less than a 50% reduction in MHD. Notably, patients with less than a 50% reduction in MHD had some reduction in their interictal burden, but this effect remained modest and insignificant. Patients with less than a 30% MHD reduction at T1 had the same degree of interictal burden, compared to T0.

BoNTA treatment was associated with significant improvements in all efficacy variables, and a significant decline (*p* < 0.001) at T1, compared to baseline, was disclosed. The mean MHD substantially declined from 21.5 ± 5.3 at T0 to 7.8 ± 5.1 at T1. At T1, the median MHD with a peak headache intensity of ≥5 and the MHD frequency with the intake of abortive medications were reduced to one third. Migraine-related disability was significantly decreased, according to the median HIT-6 scores, which dropped by −15 (*p* < 0.001) at T1, compared to T0. The changes in all efficacy and disability outcomes between T0 and T1 (median, range, z, *p* and effect size values) are summarized in Table 3.

There was a positive association between the improvement in interictal burden, assessed with MIBS-4, and a reduced MHD frequency at T1 (Pearson’s correlation: 0.677; *p* < 0.001). In contrast, the ACS-12 scoring was not associated with reduced MHD rates at T1 (Pearson’s correlation: 0.15; *p* = 0.228). 

### 2.4. Safety Analysis (Secondary Objective)

No new BoNTA-related safety signals or systemic AEs were evident. Mainly transient and mild in severity adverse events were disclosed, including neck pain (n = 10), erythema at the injection site (n = 6); mild ptosis (n = 4) and eyebrow elevation (n = 3). There were no withdrawals from the study because of side effects. 

## 3. Discussion

It has been previously demonstrated that central sensitization, clinically manifested with cutaneous allodynia in the cephalic areas of chronic migraineurs, significantly adds to both the ictal and interictal burdens of the disease [30]. We herein aimed to provide a deeper understanding of whether an adequate treatment plan with three consecutive BoNTA courses injected quarterly might have beneficial effects in alleviating the degree of both the allodynia and interictal burden. To explore our primary objectives, a well-characterized cohort of CM patients was treated with the fixed-site–fixed-dose PREEMPT strategy, pointing at the peripheral nerve endings of the cervical and trigeminal sensory systems [21,22].

The percentage of cutaneous allodynia (68.6%) in our cohort of CM patients was in line with epidemiological estimates suggesting that allodynia may account for up to 90% of chronic migraineurs [30,31,32,33,34]. However, much lower percentages, i.e., less than 20%, were reported in other studies [35,36], strongly supporting the view that methodological discrepancies may account for the significant differences in the incidence of allodynic migraineurs. In previous attempts to recognize and grade allodynia, migraineurs were evaluated using either quantitative sensory testing (thermal allodynia), which is an examination with limited use due to its limited availability [37], or a subjective questionnaire [38], including the 12-item Allodynia Symptom Checklist (ASC-12) [29]. Our patients were prospectively monitored during BoNTA treatment with the reliable ACS-12 tool to provide clinical information concerning the severity of cutaneous allodynia, whereas, to increase the accuracy of our results, we also clinically examined them to assess the severity of dynamic mechanical (brush) allodynia. We have shown that BoNTA can significantly reduce the extent of cutaneous allodynia in our CM patients, as demonstrated by the significant reduction in both ASC-12 and PI-NRS VAS hair brushing scores at the end of the observation period, compared to baseline, through the modulation of nociceptive transmission. Our results are in agreement with previous studies also showing that BoNTA seems to have beneficial effects on the central sensitization clinically expressed by cutaneous allodynia [29,39], while also supporting the view suggesting that allodynia might be a strong predictor of BoNTA efficacy in CM prophylaxis [24].

Apart from the obvious efficacy and safety of BoNTA in CM prophylaxis, which was again re-confirmed in the current real-world study, we also found that BONTA improved the interictal burden of our CM patients, as evidenced by the significant decline in MIBS-4 scoring, which decreased by a median of −7 at T1 compared to baseline, while the percentage of patients with a moderate/severe interictal burden decreased substantially after BoNTA treatment. However, a very important finding of our study was that the degree of the interictal burden was not influenced by the lessened extent of cutaneous allodynia. On the contrary, the reduced MHD frequency at T1 was significantly associated with a smaller interictal burden at the same time point. This observation advocates in favor of the view that the interictal burden in CM is a distinct effect of the disease that remains related to BoNTA-related improvements in headache efficacy variables, i.e., mostly MHD reductions and the subsequent de-escalation from chronic to episodic migraine. In addition, it seems that the interictal burden is not fully captured by other constructs, such as the improvement in cutaneous allodynia. To our knowledge, there are no similar studies focusing on the effects of BoNTA on the interictal burden of CM patients, and our results can only be indirectly compared with the results of recent studies that have evaluated changes in interictal burden with anti-calcitonin gene-related peptide (CGRP) monoclonal antibodies (anti-CGRP/anti-CGRPr MAbs) and found that the treatment with galcanezumab resulted in a significant reduction in interictal burden [28,40,41].

We should acknowledge that the observational design that we applied, and the relatively moderate sample size that was evaluated after three consecutive BONTA courses were given quarterly, might limit the generalization of our findings. Our study lacked an examination of patients with outcome measures to assess the physical and emotional well-being domains of quality of life, which may be factors allowing for a more thorough assessment of the interictal burden. However, despite these caveats and the short timeframe of the study period, we could demonstrate that improvements in interictal burden and cutaneous allodynia were observed with BoNTA, while clinically meaningful changes in all core efficacy variables, coupled with excellent safety/tolerability, were also disclosed. 

## 4. Conclusions

To conclude, adequate treatment with BoNTA, injected quarterly for three consecutive cycles, resulted in a statistically significant reduction in interictal burden and also diminished the extent of cutaneous allodynia, while the previously reported reduction in ictal burden, as evidenced by the reduction in MHD and HIT-6 scores at the end of the follow-up period, was re-confirmed. The improvements in ictal burden (within attacks) were associated with a reduction in the degree of interictal burden (between attacks). As such, BoNTA could improve the full spectrum of migraine impairment by reducing the clinical expression of central sensitization, thus significantly contributing to the inhibition of migraine chronification. Cutaneous allodynia, experienced interictally, likely predisposes patients to a transformation from HFEM to CM and, as such, BoNTA might merit assessment in HFEM patients with evidence of prominent interictal allodynia to likely tackle the escalation of MHD. In addition, further larger studies employing assessments of the many constructs of migraine disability, including physical and emotional well-being, are warranted to assess the benefits of preventative treatments for the interictal burden of migraine patients. Whether there is indeed a relationship between improvements in ictal and interictal burdens should also be further explored. 

## 5. Materials and Methods

### 5.1. Study Design and Patients’ Selection

This was a prospective, open-label study, wherein adult CM patients who were scheduled and agreed to receive BoNTA treatment, according to the approved indication and the national reimbursement policies, were enrolled and longitudinally followed up to serve its primary and secondary purposes. According to the current national policies, full reimbursement is granted for BoNTA in the setting of CM prophylaxis to patients who fail to respond or are intolerant to three previous conventional, orally given medications. The general inclusion and exclusion criteria remained the same, as previously described by our research group [42,43,44]. Briefly, the inclusion criteria were the following. (i) A definite diagnosis of CM with or without aura or MOH, according to ICHD-III criteria [5]. MOH was defined as a secondary headache presenting for at least 15 days/month in patients with a pre-existing headache disorder because of regular acute headache medication overuse for more than three months and not accounting for any other ICHD-III diagnosis [5,45]. Aura was defined as focal disturbances (neurologic, gastrointestinal and autonomic), usually occurring as visual, sensory or motor symptoms before or during a migraine attack [46]. (ii) Patients were BoNTA-naïve. (iii) Patients used BoNTA as a monotherapy for their migraine prophylaxis (no other CM oral preventatives were allowed, besides antidepressants for psychiatric comorbidities). (iv) Patients were willing to keep headache diaries and complete other migraine-related questionnaires, as instructed. (v) Patients agreed to be contacted to conduct additional phone interviewing to gather any further required data. Patients with any other major neurological condition, which might have complicated the assessments, were excluded.

BoNTA (Botox^®^ 100UI/fl, Abbvie, Athens, Greece) was administered quarterly by certified BoNTA injectors throughout the study’s period, strictly adhering to the fixed-dose–fixed-site PREEMPT paradigm [21,22], using the standard 0.5-inch 27G needle. Patients received three consecutive treatment (1 per trimester) cycles, before defining the clinical outcomes of intervention; thus, the study duration was 11 months, including two months after commencing the third BoNTA course to obtain the last efficacy follow-up. Patients withdrawing early for any reason were excluded from the formal analysis of the data, because the primary objective of the current study was not to establish the efficacy of BoNTA in CM prophylaxis, as outlined below. 

### 5.2. Primary and Secondary Objectives

Our primary objective was to evaluate the changes in the frequency and severity of interictal burden and cutaneous allodynia, from baseline (T0—3-month pre-BoNTA period) to the period of two months after the administration of the last (third) course of BoNTA (T1). The term “interictal burden” was defined as the impact of migraine on patient life in the absence of a migraine attack [47]. This outcome was quantified with the use of the Migraine Interictal Burden Scale (MIBS-4) in paper format, which is a 4-item self-report questionnaire used to measure the interictal burden in migraineurs. Each item is scored on a six-point Likert scale (don’t know/NA; never; rarely, sometimes; much of the time; and much/all the time) to eventually construct a sum score ranging from 0 to 12. Higher MIBS-4 total scores indicate the worst interictal burden, with gradings as follows: 0: no interictal burden; 1–2: mild grade; 3–4: moderate grade; and ≥5: severe level of interictal burden [48].

Cutaneous allodynia in our CM patients was defined as the presence of unpleasant or painful responses to external non-noxious stimuli, when these were applied in the cephalic area [49]. The assessment of the frequency and severity of cutaneous allodynia was performed with the use of the 12-item Allodynia Symptom Checklist (ASC-12). This tool has a sum score ranging from 0 to 24 based on responses to each item scored on a six-point Likert scale (don’t know/NA; never; rarely, sometimes; much of the time; and much/all the time). Higher ASC-12 scores are in line with the worst severity of allodynia and corresponding gradings are 0–2: no allodynia; 3–5: mild allodynia; 6–8: moderate allodynia; and ≥9: severe allodynia [50]. The severity of cutaneous allodynia was clinically quantified by asking patients to brush their hair and report the degree of discomfort based on the PI-NRS score, which is an 11-point pain intensity numerical rating scale ranging from 0 = no pain to 10 = worst possible pain [51]. For the current study, the effects of BoNTA on the interictal burden and allodynia were assessed by estimating the changes in the corresponding frequencies and severities, according to MIBS-4, ACS-12 and PI-NRS (hair brushing) scorings, from T0 to T1.

Secondary objectives included (i) the estimation of changes in the BoNTA-related response rates, defined as an at least 50% reduction in mean monthly headache days (MHD) at T1, compared to T0; the crude percentage of patients experiencing a < or >50% reduction in their MHD was analyzed; (ii) the change in migraine severity as measured by the change in mean MHD with peak moderate/severe headache intensity, i.e., at least 5 (≥5) out of 10 in a 0–10 visual numerical scale, from T0 to T1; (iii) the change in mean MHD with the use of medications for acute migraine headache between T0 and T1; and (iv) the documentation of changes in headache-related disability, as measured by the 6-item Headache Impact Test (HIT-6), from T0 to T1. The HIT-6 measures the impact of migraine on domains such as pain, social functioning, role functioning, vitality, cognitive functioning and psychological distress, based on the patients’ responses (“never”, “rarely”, “sometimes”, “very often” or “always”), to eventually construct a sum score that ranges from 36 to 78. A higher HIT-6 score indicates greater migraine-related disability [52]. 

### 5.3. BoNTA Safety Evaluation

The evaluation of safety was included among our secondary objectives and was performed by asking patients to report any adverse events on days 1–14 following every BoNTA infusion. The patients’ reported adverse events were then evaluated for a potential relationship with BoNTA. 

### 5.4. Statistical Analysis

SPSS for Windows (release 27.0; SPSS Inc., Chicago, IL, USA) provided descriptive statistics for all variables depending on their nature. The Wilcoxon rank test for paired data was used to document changes in the values (mean or median, range, z, *p* and effect size values) of primary and secondary efficacy variables, between T0 and T1. Associations were made by computing the Pearson’s correlation coefficient. Unless otherwise stated, all tests were two-sided, and significance was set at *p* < 0.05. 

## Figures and Tables

**Table 1:** Demographic and clinical characteristics of participants at baseline.

Participants n = 70	
Variable	N (%)
**Gender**	
Females	65 (92.9)
Males	5 (7.1)
**Age ± SD (range)**	44.4 ± 9.8 (21–68)
**Previous lines of prophylactic medications**—Median value (range)	4 (3–6)
**Years with migraine diagnosis**—Median value (range)	16 (2–44)
**Psychiatric comorbidities**	
No	30 (42.9)
Anxiety disorder	19 (27.1)
Depression	12 (17.1)
Mixed anxiety and depression disorder	6 (8.6)
Bipolar disorder	3 (4.3)
**Medication overuse headache**	
Yes	58 (82.6)
No	12 (17.4)
**Aura**	
Yes	7 (10.0)
No	63 (90.0)

**Table 2:** Changes in interictal burden and allodynia outcomes at baseline (T0—trimester before initiation of therapy) and after 3 courses of BoNTA treatment (T1) in enrolled patients with chronic migraine (n = 70).

Median (Minimum–Maximum)					
Variables	Baseline	After Treatment with 3 BoNTA Cycles	z	*p* Value	Effect Size
MIBS-4 score	9 4–12	2 0–10	−7.222	<0.001	0.86
ASC-12 score	6 0–13	1 0–12	−5.393	<0.001	0.65
PI-NRS for hair brushing	5 0–9	1 0–8	−5.398	<0.001	0.65

**Table 3:** Responder rates and changes in efficacy and disability outcomes at baseline (T0—trimester before initiation of therapy) and after 3 courses of BoNTA treatment (T1) in enrolled patients with chronic migraine (n = 70).

Median (Minimum–Maximum)					
Variables	Baseline	After Treatment with 3 BoNTA Cycles	z	*p* Value	Effect Size
Monthly headache days	22 15–30	5 2–19	−7.277	<0.001	0.87
Monthly days with peak headache intensity of at least 5/10 in VAS	10 4–30	3 1–18	−7.262	<0.001	0.86
Monthly days with intake of acute headache medication	12 5–30	4 1–19	−7.172	<0.001	0.85
HIT-6 score	71 56–78	56 48–66	−6.962	<0.001	0.83
Response to treatment (%)					
<30%		5 (7.0)			
>30–<50%		11 (15.5)			
>50–<75%		35 (49.3)			
>75–<100%		19 (26.8)			
100%		0 (0)			

## Data Availability

The data that support the findings of this study are available from the corresponding author upon reasonable request.
